# Microcystic/Reticular Schwannoma of the Frontal Lobe: An Unusual Occurrence

**DOI:** 10.1155/2017/4728585

**Published:** 2017-03-27

**Authors:** Lauren Pearson, Erinc Akture, Julien Wonderlick, Gregory Fuller, Maryam Zenali

**Affiliations:** ^1^Department of Pathology, University of Vermont Medical Center, Burlington, VT, USA; ^2^Department of Neurosurgery, University of Vermont Medical Center, Burlington, VT, USA; ^3^Department of Radiology, University of Vermont Medical Center, Burlington, VT, USA; ^4^Department of Pathology, University of Texas MD Anderson Cancer Center, Houston, TX, USA

## Abstract

Schwannoma is a benign peripheral nerve sheath tumor that typically involves cranial nerves of the head and neck region. Intraparenchymal occurrence of this tumor is uncommon. Even rarer in this site is the microcystic/reticular pattern of schwannoma. This histologic variant, first described in 2008, has a predilection for visceral organs. Herein, we report the first case of microcystic/reticular schwannoma of the frontal lobe.

## 1. Introduction

Schwannomas are benign tumors of the nerve sheath, typically solitary and most commonly involving the head and neck region. Classic schwannomas are histologically characterized by two distinct patterns: cellular spindle foci with nuclear palisading (Antoni A) alternating with more hypocellular areas (Antoni B). Different histologic forms of schwannomas include plexiform, psammomatous, melanotic, and so-called ancient. A more recently identified variant is the microcystic/reticular schwannoma, described in 2008 [1–3].

Intraparenchymal occurrence of schwannoma is uncommon and first reported in 1966 by Gibson et al. [4] Since then, greater than sixty-five cases of intracerebral schwannomas (IS) have been described [4–28]. Schwannomas can also occur in the viscera, with the gastrointestinal tract being the most common site. The newly described microcystic/reticular variant (MRV) has a tendency to occur in the viscera [29–34].

In the original publication of the microcystic/reticular variant [2], a total of ten cases were described, amongst which half involved the gastrointestinal tract. The remaining five cases occurred in the following sites: respiratory tract, adrenal gland, extremities, and posterior trunk. MRV was described as having a striking microcystic and reticular growth pattern, anastomosing and intersecting spindle cells with eosinophilic cytoplasm, set in a collagenous to myxoid stroma. All of these lesions lacked a capsule and were S-100 positive. Although the microcystic/reticular pattern was the predominant morphology, histology varied with respect to the presence and extent of the more classic features.

In this report, we review the histopathology and clinical features of MRV schwannoma involving the left frontal lobe of a 22-year-old woman.

## 2. Case Report

### 2.1. History and Examination

**Figure 1:**
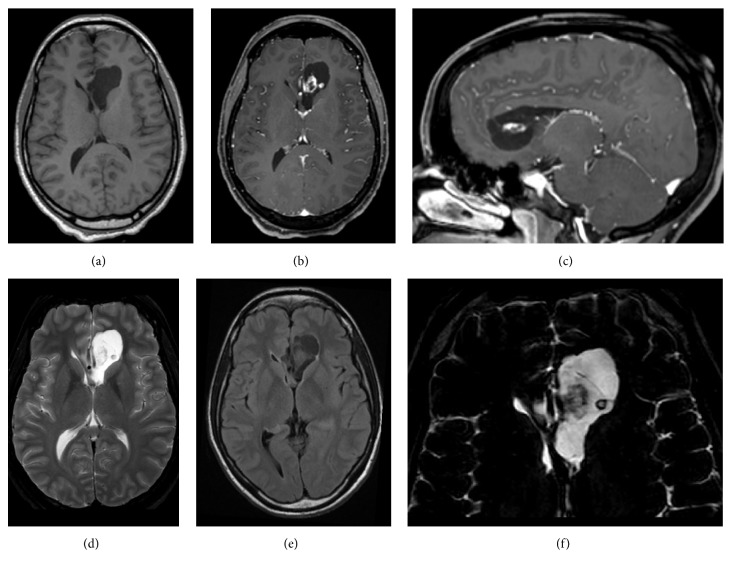
Precontrast axial (a), postcontrast axial (b), and postcontrast left parasagittal (c) T1-weighted MR images of the brain demonstrate an intra-axial left frontal mass with heterogeneous internal nodular and septal enhancement. The largest enhancing component is subjacent to markedly thinned medial left frontal cortex. T2-weighted axial images (d) demonstrate hyperintense cystic formation with near complete suppression on FLAIR sequences (e). No peritumoral T2 hyperintensity is present to suggest vasogenic edema or gliosis. Details from high-resolution, heavily T2-weighted images (f) redemonstrate mixed solid and cystic components and thin internal septation. No elevated perfusion or abnormally restricted diffusion was identified on MR perfusion or diffusion-weighted imaging, respectively (not shown).

A 22-year-old woman presented to the neurosurgery clinic with a 3-month history of progressive headaches. There was no significant past medical history, including no patient or family history of neurofibromatosis and no other known genetic disorder. The patient was otherwise healthy. Magnetic resonance imaging (MRI) of the brain demonstrated a mixed cystic and solid, heterogeneously enhancing intra-axial mass centered within the left frontal lobe effacing the adjacent frontal horn of the lateral ventricle (Figure 1). Notably, no T2 hyperintense perilesional edema was present. No abnormal MR perfusion was associated with enhancing portions of the mass, and no abnormally restricted diffusion was evident on diffusion-weighted sequences. There was no evidence of hemorrhage or calcification on susceptibility-weighted MR images or on supplemental computed tomography (CT) imaging. Initial differential considerations included supratentorial pilocytic astrocytoma or other low-grade astrocytomas, atypical ependymoma, or less likely central neurocytoma given possible extension into the lateral ventricle. The patient was neurologically intact and was lost to follow up for two years after the initial presentation. She then returned to the neurosurgery clinic with headaches and personality change. Repeat MRI of the brain demonstrated interval increase in the size of the mass to 3.7 cm from 3.0 cm in maximum dimension; the imaging appearance of the mass was otherwise stable. A decision for microsurgical resection was made. The patient underwent a bifrontal craniotomy with frameless stereotactic guidance. Gross total resection was achieved. The patient tolerated the surgery well and was discharged home without any complication.

### 2.2. Pathology

The specimen consisted of three tan-white hemorrhagic and irregular soft tissue fragments (0.5 grams, 1.8 × 1.2 × 0.6 cm in aggregate). Squash preparation, frozen section, and formalin fixed paraffin embedded tissues were evaluated histologically.

**Figure 2:**
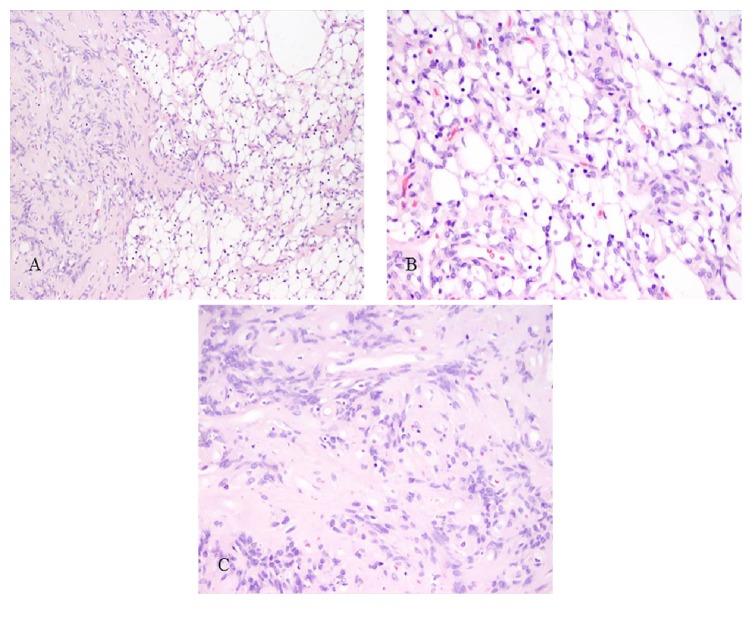
Photomicrographs of hematoxylin and eosin sections: (A) microcystic/reticular foci with a myxoid appearance adjacent to denser spindle cell areas (100x magnification); (B) microcystic areas with vacuolated cells; and (C) bland spindle cells with palisading configurations (B&C: 400x magnification).

The lesion was unencapsulated and composed of microcystic foci with small nuclei and vacuolated cytoplasm, set in a myxoid-appearing stroma. There were intervening confluences of spindle cells with ovoid nuclei and eosinophilic cytoplasm. Occasional hypercellular foci with palisading growth pattern were present. There was no necrosis, significant cytologic atypia, or mitoses (Figure 2).

**Figure 3:**
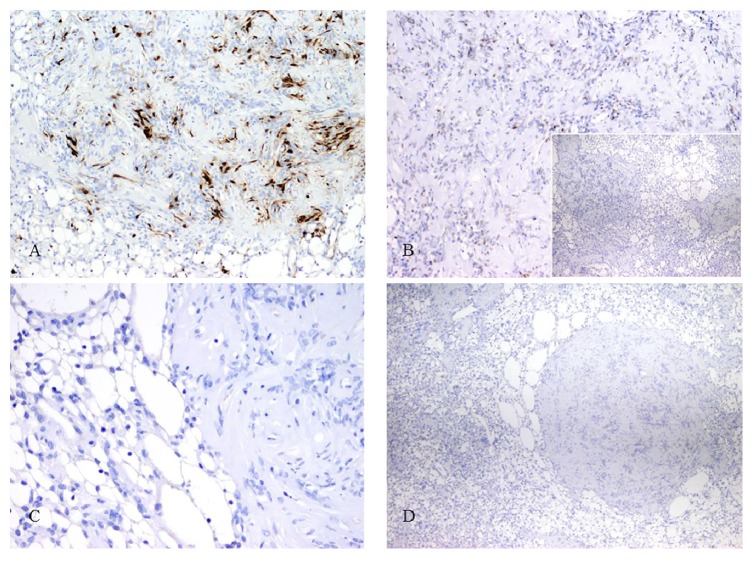
Photomicrographs of immunohistochemical stains: (A) S-100 (400x): nuclear and cytoplasmic staining in tumor cells, (B) progesterone receptor (400x): focal staining and (B inset) estrogen receptor (100x): no staining in tumor, and (C) GFAP (400x) and (D) EMA (100x): negative staining in tumor.

A Periodic Acid-Schiff stain was negative in the vacuolated cells. Tumor cells were positive for S-100 (DAB, Thermo Scientific 4C4.9) in their nuclei and cytoplasm and had patchy staining with CD34 (QEnd/10) and calretinin (cal6) and focal staining with progesterone receptor (PR1294, Dako) immunostain. Tumor cells were negative for estrogen (ID5), EMA (E29, Dako), Bcl-2 (mouse monoclonal 124), CD10 (56C6, Thermo Scientific), inhibin (R1, Thermo Scientific), and GFAP (polyclonal, Dako) immunostains (Figure 3).

## 3. Discussion

Intracerebral schwannomas are generally diagnosed in children or young adults, commonly presenting with headaches or seizures [5–8, 23, 28]. Depending on their location in the central nervous system (CNS), they may manifest with focal neurologic damage [5, 7]. While in children they are typically slow growing and indolent, in the older patients, they can grow rapidly and present with neurologic deficits stemming from increased cranial pressure or compression of adjacent structures [5–8]. The projected clinical behavior of these lesions was demonstrated by our patient who initially presented with headaches and later developed personality changes as the tumor increased in size.

Schwannomas are benign lesions, typically well-circumscribed with a collagenous capsule. Classic schwannomas are composed of compact spindle (Antoni A) and hypocellular spindle (Antoni B) areas with scattered palisading nuclei known as Verocay bodies. Most lesions are sporadic, although a proportion is associated with neurofibromatosis, schwannomatosis, and Carney complex [3]. In adults, schwannomas represent 8% of primary brain tumors and most commonly arise from the vestibular nerve [35]. Involvement of the brain parenchyma and spinal cord is rare [3]. In these locations, schwannomas can resemble other more common intra-axial cystic neoplasms including pilocytic astrocytoma, pleomorphic xanthoastrocytoma [7], meningioma if near a centrally located nerve root [9, 10, 13, 14], or less commonly a high-grade glial tumor [11].

The origin of intraparenchymal schwannoma is much debated in the literature. There are two theories for their occurrence in the central nervous system. One is that they have a developmental origin related to native CNS cells such as the mesenchymal pial cells or neural crest cells. Another theory attributes the presence of Schwann cells in the brain to the perivascular nerve plexuses and the large arteries in the subarachnoid spaces [5, 36].

Due to rare intracerebral occurrence and lack of pathognomonic imaging features, intraparenchymal schwannoma may be overlooked in the initial differential diagnosis of an intra-axial tumor. Several characteristic findings have been described, although significant variability exists. These lesions are often superficial or periventricular in location and may be enhanced homogenously or heterogeneously [7, 37]. Cyst formation is commonly described, evident as fluid attenuation on CT and T1 hypointense, T2 hyperintense signal on MR images which may be partially or completely suppressed on FLAIR imaging [5, 8, 37]. Macroscopic calcification is sometimes seen on CT images and gradient echo (GRE) or susceptibility-weighted MR imaging. Notably, calcification is rare and cyst formation uncommon in vestibular schwannoma [37]. Peritumoral vasogenic edema is present in a majority of reported cases of IS [7, 37]. In this case, the lesion was periventricular in location with heterogeneous enhancement and cyst formation but lacked imaging evidence of calcification or peritumoral edema.

In contrast to other variants of schwannomas, MRV lacks a capsule and most importantly has a predilection for visceral locations [29–33]. To the best of our knowledge, this case is the first reported reticular/microcystic variant in an intracerebral location. This tumor manifested radiologically as a mixed cystic and solid enhancing intra-axial mass. Histologically, it was characterized by anastomosing and intersecting spindle cells admixed with vacuolated cells within a collagenous to myxoid stroma; scattered Verocay bodies were evident.

While there is some variability in histopathologic description of MRV schwannoma, common features include microcystic/reticular spaces and myxoid material. Tumor cells are spindle to vacuolated cells and have ovoid nuclei, inconspicuous nucleoli, and eosinophilic cytoplasm. Foci with classical schwannoma patterns (Antoni A and Antoni B areas) are often present. Lymphoid aggregates can be seen in a subset, for instance, in the soft tissue tumors. Nuclear atypia, necrosis, and mitotic activity are rare or absent [2, 8–39].

Despite their distinct appearance, thus far MRV lesions appear to behave similar to classic schwannomas, with no recurrences reported in the literature after excision. These tumors are positive for cytoplasmic and nuclear S-100 and are negative for EMA, Pan-CK, desmin, and Bcl-2 in the tumor core. They can have focal staining with GFAP and calretinin [2, 38, 39].

Our case is the first intracerebral microcystic/reticular schwannoma reported in the literature. Care must be taken to include this entity in the differential diagnosis of a mixed cystic and solid brain lesion. Prospective imaging diagnosis is challenging due to a lack of pathognomonic features; however, characteristic imaging findings can support a pathologic diagnosis. Our patient is doing well three years after the surgery with no residual neurologic deficit.
